# Engineered α‐Helical Peptides with Chelating Agents as Approach to Antibacterial Therapeutics

**DOI:** 10.1002/open.202500588

**Published:** 2026-01-08

**Authors:** Vincenzo Patamia, Erika Saccullo, Michele Larocca, Virginia Fuochi, Salvatore Furnari, Pio Maria Furneri, Agostino Cilibrizzi, Giuseppe Floresta

**Affiliations:** ^1^ Department of Drug and Health Sciences University of Catania Catania Italy; ^2^ Department of Biomedical and Biotechnological Sciences (Biometec) University of Catania Catania Italy; ^3^ Department of Chemistry and Biology “Adolfo Zambelli” University of Salerno Fisciano (Salerno) Italy; ^4^ Institute of Pharmaceutical Science King's College London London UK; ^5^ Centre for Therapeutic Innovation University of Bath Bath UK

**Keywords:** allomaltol, antibacterial, chelating agents, main mechanical forces, peptides

## Abstract

This study introduces a new class of α‐helical antimicrobial peptides designed to combat multidrug‐resistant bacteria. The peptides were created using a structure‐based approach guided by the main mechanical forces (MMFs) methodology, which promotes stable helical conformations by considering chemical interactions between amino acid side chains. Key features of the design of these peptides include: (1) amphipathic nature: hydrophobic and cationic residues are strategically positioned on opposite sides of the helix to disrupt bacterial membranes and (2) MMFs approach: enables precise control over the peptide's 3D structure through dihedral angle calculation. The peptides exhibited antimicrobial activity against various bacterial strains, including both Gram‐positive and Gram‐negative species, as well as a multidrug‐resistant pathogen. This effect was particularly enhanced when coadministered with allomaltol, a chelating agent capable of sequestering essential metals (such as iron), thereby disrupting bacterial metabolism and providing a secondary mechanism of action. This work validates the MMFs methodology as an accurate prediction tool for peptide secondary structure, reproducing NMR‐derived helical features of the HT2 peptide and enabling rational design of new analogs. Moreover, the covalent introduction of a chelating group markedly improved antimicrobial potency (MIC 18.75 μM vs. 300 μM), confirming the functional synergy between amphipathic helicity and metal‐ion sequestration.

## Graphical abstract

This study introduces a new class of α‐helical antimicrobial peptides rationally designed using the main mechanical forces. The peptides exhibit broad‐spectrum antimicrobial activity against Gram‐positive, Gram‐negative, and multidrug‐resistant pathogen strains. Efficacy is significantly enhanced by allomaltol coadministration and metal‐ion sequestration, confirmed by the potent effect of covalently introducing a chelating group (MIC 18.75 μM).

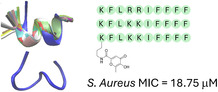

## 1 Introduction

The relentless rise of multidrug‐resistant bacteria presents an increasingly dire threat to global public health, necessitating an urgent paradigm shift in the development of novel antimicrobial therapeutics [1]. Traditional antibiotics, which typically target specific bacterial metabolic processes, are becoming progressively less effective due to the rapid emergence and spread of sophisticated resistance mechanisms [2, 3, 4, 5, 6, 7, 8]. In this context, antimicrobial peptides (AMPs), and in particular, those adopting an α‐helical conformation, have garnered significant attention as a promising alternative strategy for combating bacterial infections [4, 9, 10]. These peptides are characterized by their ability to directly disrupt bacterial membranes, leading to rapid cell death, a mechanism less prone to the development of resistance than conventional antibiotics [11]. Furthermore, α‐helices are commonly found in protein–protein interaction interfaces, making them attractive templates for designing targeted therapeutics [12, 13].

While naturally occurring AMPs often demonstrate potent antimicrobial activity, their clinical translation is frequently hindered by limitations including high production costs, susceptibility to enzymatic degradation, poor bioavailability, and potential immunogenicity [11]. This has driven considerable research efforts toward the rational design and synthesis of novel AMPs, particularly those with enhanced efficacy, stability, and improved physicochemical properties [11]. The rational design of synthetic α‐helical peptides offer a compelling approach to circumvent the limitations inherent in natural AMPs [11]. This process involves the strategic creation of peptides with specific structural features, such as a short sequence (typically 5–50 amino acids), a net positive charge, and an amphiphilic character, all of which are crucial for their antimicrobial activity. Key design strategies encompass template modification, minimalist *de novo* design, combinatorial library technology and in silico modeling [11].

This paper reports on the design, synthesis, and evaluation of novel α‐helical peptides engineered to exhibit broad‐spectrum antibacterial activity. These peptides were meticulously designed using a structure‐based approach that leverages the main mechanical forces (MMFs) methodology to guide the formation of stable and functional α‐helical conformations [14, 15]. The MMF approach, which considers the specific chemical interactions between the side chains of adjacent amino acid residues, provides a powerful tool for calculating the dihedral angles that dictate the peptide's 3D structure and stability, ensuring the desired helical conformation is achieved [15]. This innovative approach allows for precise control over the peptide's conformation, optimizing its interaction with target biological structures, such as membranes in the context of this research. In contrast to most structure‐based peptide design studies, our primary aim was to validate the predictive reliability of the MMF approach by benchmarking it against NMR‐derived conformers of the reference peptide HT2. This represents the first quantitative validation of the MMF method for α‐helical AMPs, thereby bridging theoretical modeling and experimental spectroscopy.

Moreover, the designed peptides incorporate an amphipathic architecture, strategically positioning hydrophobic and cationic residues on opposing faces of the *α*‐helix. This spatial arrangement facilitates the peptides’ insertion into and interaction with the lipid bilayer of bacterial cell membranes, leading to membrane permeabilization and subsequent cell death [4]. Beyond this essential membrane‐disrupting function, the activities of the peptides are further enhanced by the coadministration of a chelating agent. This further strategy is conceived to benefit a secondary mechanism of antimicrobial action, based on the sequestration of essential metal ions (such as iron) from the bacterial environment [16], thereby disrupting critical metabolic processes and further compromising cell viability [3]. This dual mechanism is anticipated to enhance the peptides’ overall efficacy and reduce the likelihood of resistance development. In parallel with validating the MMFs approach, we also explored an orthogonal synergistic axis in which an amphipathic α‐helical scaffold is covalently linked to a metal‐chelating moiety. Iron deprivation is recognized as a potent enhancer of antimicrobial activity, as demonstrated for synthetic chelators that potentiate antibiotics and AMPs [16, 17, 18]. This dual‐mechanism concept supports the well‐established framework of nutritional immunity, in which host‐driven metal sequestration suppresses bacterial growth [19].

The findings of this study demonstrate that these rationally designed peptides exhibit antimicrobial activity against a range of bacterial strains, including both Gram‐positive and Gram‐negative species, and importantly, multidrug‐resistant clinical isolates [4, 11]. This activity is attributable to the combined effects of bacterial membrane disruption, driven by the amphipathic α‐helical structure, and the disruption of bacterial metabolism via the metal‐chelating function. Such combined effects are crucial to overcome resistance mechanisms. The promising, but not exceptional, results of this study indicate the potential of these new multifunctional peptides as therapeutic agents with good applicability in the fight against bacterial infections, particularly those caused by drug‐resistant pathogens. Combining powerful membrane disruption and metal ion chelation mechanisms, this newly developed class of peptides shows good potential for future development of antimicrobial drugs, but requires significant improvement [20]. Furthermore, the application of the MMF approach demonstrates the suitability of this methodology as a good tool for the analysis and rational design of peptides with predictable and stable conformations. While previous studies focused on activity optimization, the work herein primarily methodological and provides the first quantitative validation that MMF calculations can reproduce the experimental secondary structures of AMPs. Furthermore, the presence of a covalently linked chelating agent within the peptide structures demonstrates that the combination of structural and mechanistic design principles has good potential, which, however, needs to be improved, for example, by modifying the synthetic approach with the insertion of appropriate spacers to increase the synergistic effect of the two portions.

## 2 Results and Discussion

HT2 is a short peptide with an amphipathic α‐helical head and an aromatic tail. A structure–activity relationship study of “tadpole‐like” temporin‐SHf and its analogs revealed that increasing the number of aromatic residues in the tail, introducing Arg to the *α*‐helical head, and rearranging the peptide topology dramatically increased the antimicrobial activity [4].

**FIGURE 1:**
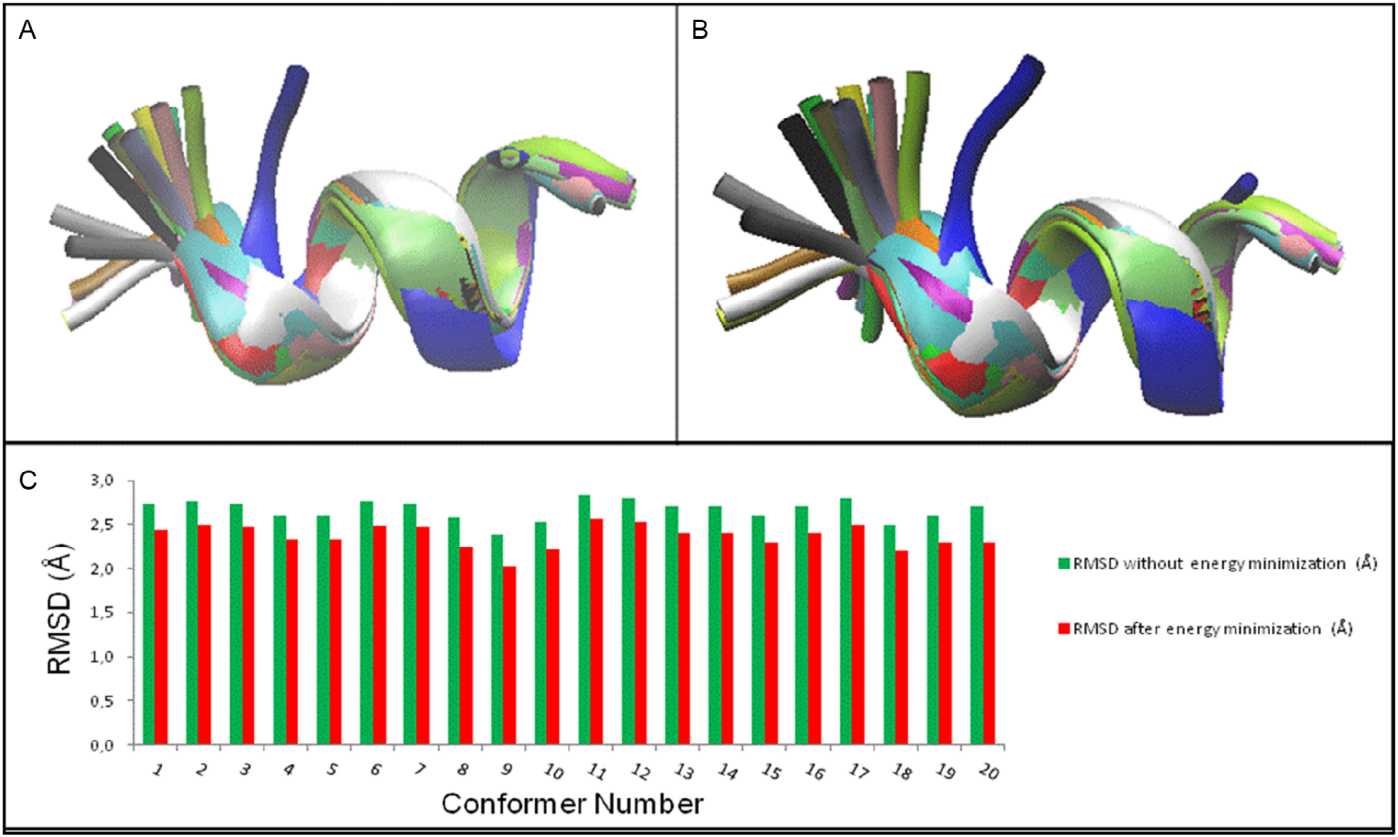
(A) Superimposition between the HT2 calculated structure without energy minimization and all the 20 NMR conformers of HT2 peptide: blue = calculated HT2, red = conformer 1, gray = conformer 2, orange = conformer 3, yellow = conformer 4, tan = conformer 5, silver = conformer 6, green = conformer 7, white = conformer 8, pink = conformer 9, cyan = conformer 10, purple = conformer 11, lime = conformer 12, mauve = conformer 13, ocher = conformer 14, ice‐blue = conformer 15, black = conformer 16, yellow 2 = conformer 17, yellow 3 = conformer 18, green 2 = conformer 19, green 3 = conformer 20. (B) Superimposition between the HT2 calculated structure with energy minimization and all the 20 NMR conformers of HT2 peptide: blue = calculated HT2, red = conformer 1, gray = conformer 2, orange = conformer 3, yellow = conformer 4, tan = conformer 5, silver = conformer 6, green = conformer 7, white = conformer 8, pink = conformer 9, cyan = conformer 10, purple = conformer 11, lime = conformer 12, mauve = conformer 13, ocher = conformer 14, ice‐blue = conformer 15, black = conformer 16, yellow 2 = conformer 17, yellow 3 = conformer 18, green 2 = conformer 19, green 3 = conformer 20. (C) RMSD values of the HT2 calculated structure with all the 20 conformers: green = without energy minimization and red = with energy minimization. Colors in A and B are those reported in visual molecular dynamics [25].

We have used the peptide HT2 as reference to carry out a structural investigation using the main mechanical forces (MMFs) approach [14, 15, 21, 22, 23] in order to figure out whether it would be possible to design new AMPs able to exhibit similar or improved features compared to HT2. Initially, the HT2 structure was calculated/predicted using the MMF approach; focusing on the need to retain the structural and chemical features of the experimental and calculated structures of HT2, we have expanded the chemical scope of this investigation by designing three structural analogs (also by means of the MMF approach), in order to test and assess their biological behavior as antimicrobial agents. The recently reported calculation methodologies [14, 15], were used to investigate the HT2 amino acid sequence theoretically: we adopted as reference the experimental conformer 1, since this was reported as the most representative within the NMR ensemble [4], and we performed MMFs calculations. Subsequently the calculated structure was compared to all the conformers (in order to determine the root mean square deviation (RMSD)) and then submitted to energy minimization using the web server Chiron [24]. Therefore, each RMSD was determined comparing the minimized calculated structure with all the 20 NMR conformers reported in literature [4]. Only the calculated structure, when compared to all the 20 conformers, exhibits a certain degree of variation, that is essentially directed toward the first two residues. In particular, these latter (ARG1 and PHE2) demonstrate to possess a very large mobility, as resulted also from the NMR analysis [9, 10], having their backbone dihedral angle averaged values with a very large standard deviation (e.g. ARG1 with *ψ*°_1_ = 64°± 74° and PHE2 with *φ*°_2_ = −90° ± 72° and *ψ*°_2_ = 74° ± 82° ‐ i.e. averaged values over the 20 NMR conformers). This feature leads to RMSD values higher than 2.0 Å when comparing all the 20 conformers to the calculated and not minimized structure (Figure 1A,C). However, the best match was obtained with the conformer 9, with an RMSD of 2.0 Å (Figure 1B,C) after energy minimization of the HT2 calculated structure. These RMSD values arise from the large mobility of the residues in position 1 and 2, as already stated above and in relation to the NMR ensemble. However, an RMSD equal to 2.0 Å was achieved with conformer 9. This enabled us to consider our calculation methodology as reliable and substantially valid to predict the HT2 structure with a substantialy high agreement. This agreement demonstrates that MMFs can reproduce experimental secondary structure distributions of short AMPs with near‐NMR accuracy. Such validation highlights the method's potential as a computationally efficient alternative to traditional molecular dynamics or over‐elaborated density functional theory (DFT)‐based conformational analyses.

**FIGURE 2:**
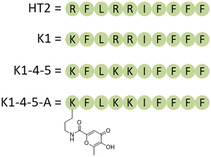
Structures of the designed peptides (K1, K1‐4‐5 and K1‐4‐5‐A) and HT2.

Once having defined the structural features of HT2 through the MMF approach, we moved forward attempting to design some analogs which were expected to have antimicrobial properties, by reproducing the HT2 biological activity. To this purpose, we applied again our recently reported MMF calculation methodologies [14, 15, 21, 22, 23]. Initially, we have decided to mimic the HT2 activity by developing an analog that retains its chemical properties, namely the derivative K1 (Figure 2) where only the arginine in position 1 is replaced with a lysine.

**FIGURE 3:**
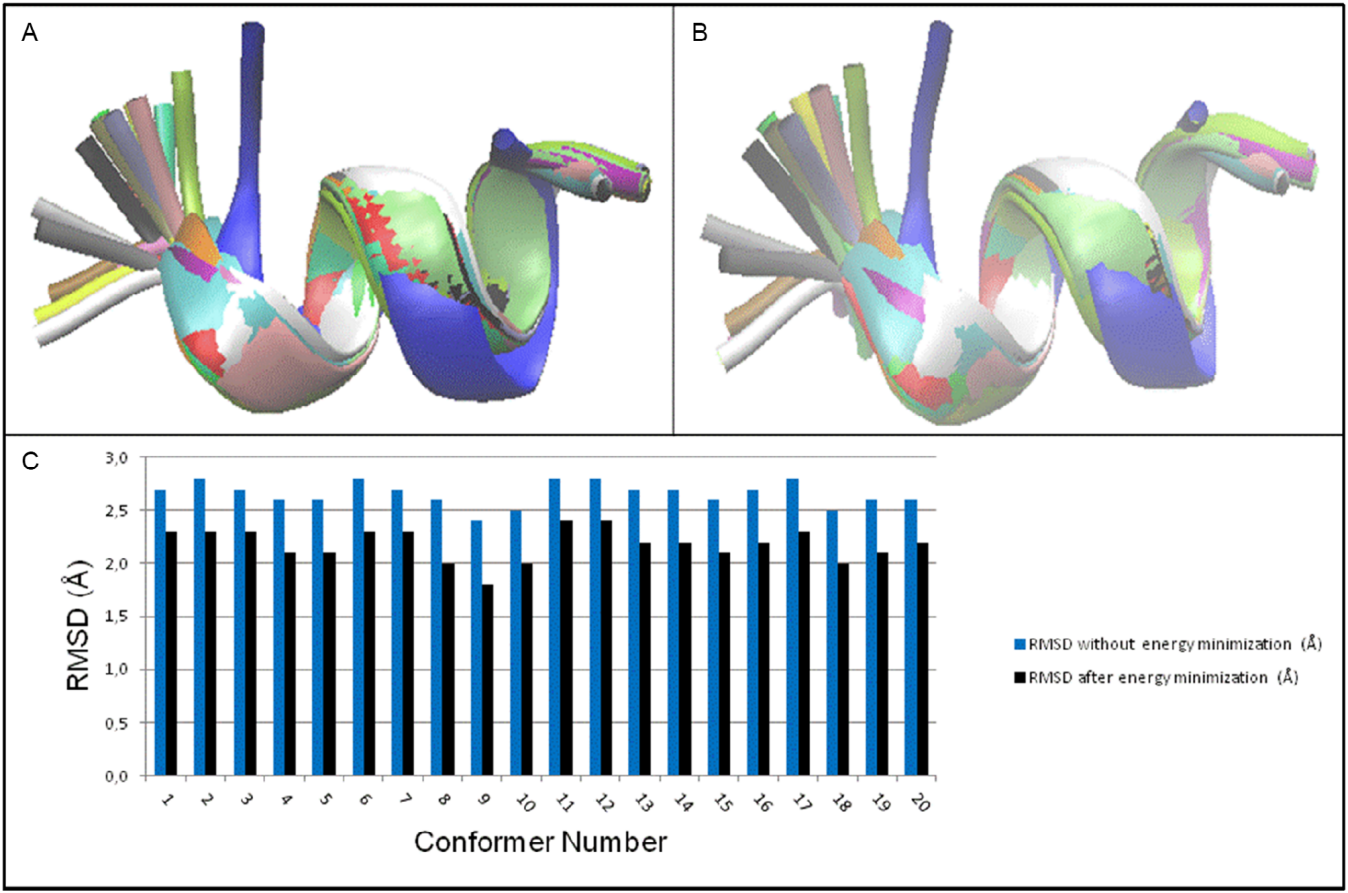
(A) Superimposition between the K1 analog‐calculated structure without energy minimization and all the 20 NMR conformers of HT2 peptide: blue = calculated K1, red = conformer 1, gray = conformer 2, orange = conformer 3, yellow = conformer 4, tan = conformer 5, silver = conformer 6, green = conformer 7, white = conformer 8, pink = conformer 9, cyan = conformer 10, purple = conformer 11, lime = conformer 12, mauve = conformer 13, ocher = conformer 14, iceblue = conformer 15, black = conformer 16, yellow 2 = conformer 17, yellow 3 = conformer 18, green 2 = conformer 19, green 3 = conformer 20. (B) Superimposition between the K1 calculated structure with energy minimization and all the 20 NMR conformers of HT2 peptide: blue = calculated K1 after energy minimization, red = conformer 1, gray = conformer 2, orange = conformer 3, yellow = conformer 4, tan = conformer 5, silver = conformer 6, green = conformer 7, white = conformer 8, pink = conformer 9, cyan = conformer 10, purple = conformer 11, lime = conformer 12, mauve = conformer 13, ocher = conformer 14, iceblue = conformer 15, black = conformer 16, yellow 2 = conformer 17, yellow 3 = conformer 18, green 2 = conformer 19, green 3 = conformer 20. (C) RMSD values of the K1 calculated structure with all the 20 conformers: blue = without energy minimization and black = with energy minimization. Colors in A and B are those reported in visual molecular dynamics [25].

Based on the literature evidence that a positive charge in position 1 is fundamental for the antibacterial activity [4], in the first instance, we have decided to maintain this chemical feature, namely a protonated amino acid residue, and replace the arginine 1 with a lysine, to further decipher (via fourier transform infrared spectroscopy (FTIR) analysis) whether the structure is retained correctly, thereby producing the expected antimicrobial activity. We have studied the K1 sequence using the recently reported MMF calculation methodology [14, 15] and analyzed its structure by comparison, before and after energy minimization, with all the 20 NMR conformers reported for HT2 (Figure 3A,B). By introducing a lysine in position 1, we obtained an improvement in terms of RMSD as demonstrated by the results for K1 compared to all the conformers after energy minimization (Figure 3A,B). Moreover, we found that the RMSD arising from the comparison with conformer 9 reached the value of 1.8 Å (Figure 3C).

Subsequently, we have studied the analog number 2 that we named K1‐4‐5 where we have replaced all the three arginines in position 1, 4, and 5 respectively with lysines. We have provided a detailed structural analysis as for K1 and report that the structure obtained exhibit a helix‐like general organization even if it does not properly match a canonical helix secondary structure.

**FIGURE 4:**
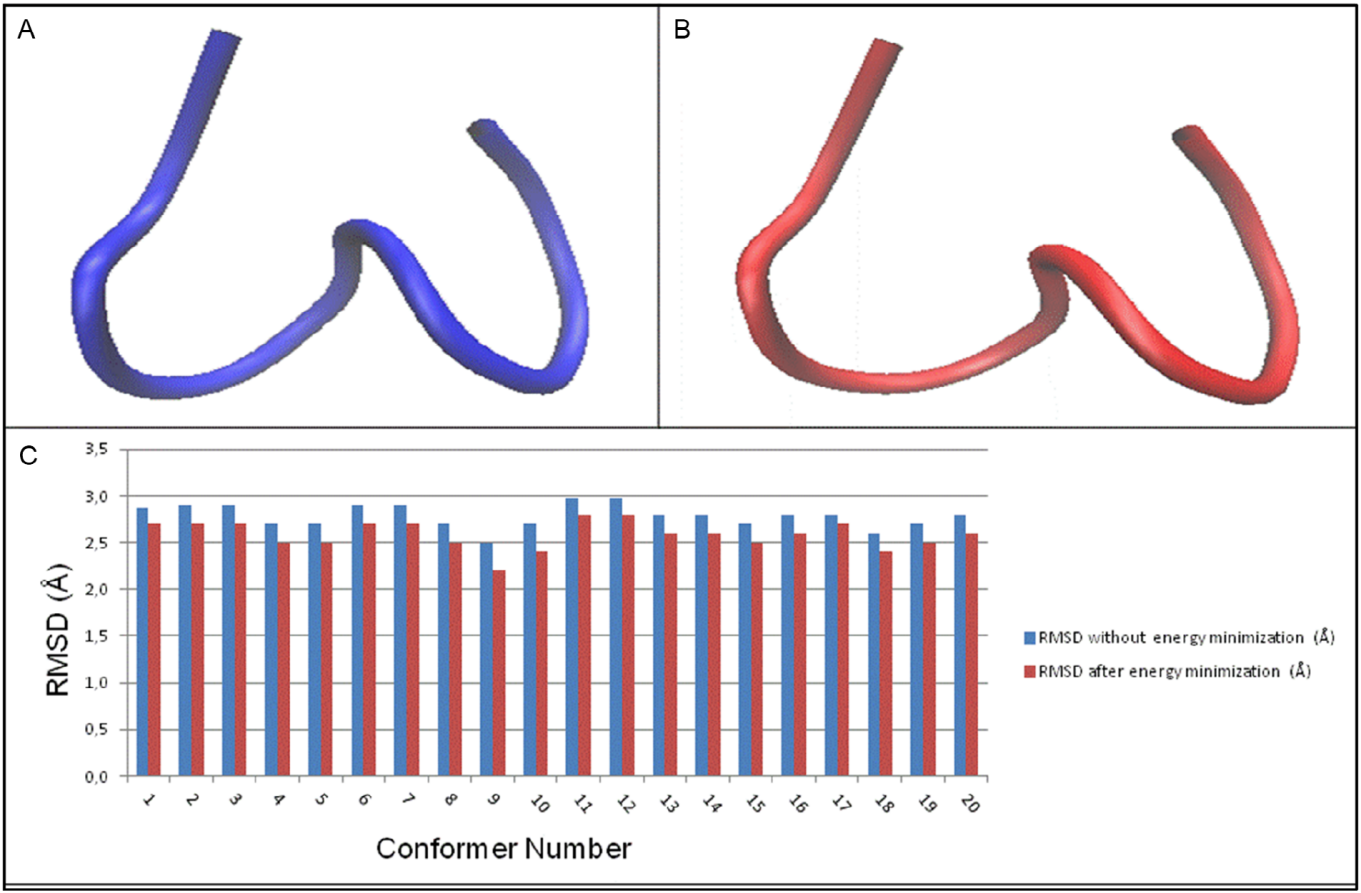
(A) K1‐4‐5 before energy minimization; (B) K1‐4‐5 after energy minimization; (C) RMSD values.

After energy minimization (Figure 4B), the K1‐4‐5 sequence was analyzed to confirm the structural features enabling to describe the general spatial organization, considering the above mentioned evidence that this structure does not exactly match a canonical helix (e.g. *α*‐helix or *π*‐helix). The Ramachandran plot of the minimized structure provides information about the distribution of the dihedral angles of each residue. From this we have found that the values of *φ*_2_, *ψ*_2,_ and *φ*_3_ adopt a β‐turn VIb structure [26], whereas *φ*_6_, *ψ*_6,_ and *φ*_7_ and *φ*_9_, *ψ*_9,_ and *φ*_10_ are consistent with a β‐turn III structure [26]. This indicates that nine dihedral angle values out of 18 (50.0%) are in line with a β‐turn structure, hence in perfect agreement with the attenuated total reflectance (ATR)‐FTIR output (Figure 6). On the other hand, a β‐sheet structure is linked to *ψ*_1_, *φ*_5_, *φ*_9_, *ψ*_9_, and *φ*_10_, namely 5 out of 18 dihedral angles values (27.8%) and the remaining values (22.2%) do not exhibit a typical secondary structure profile. Also these results are substantially in line with the ATR‐FTIR output (Figure 6).

Again, also in this case, the best match with the conformers of HT2 was obtained with conformer 9 (RMSD = 2.2 Å) when comparing K1‐4‐5 after energy minimization to all the 20 NMR conformers of HT2. Figure 3 reports the structure of K1‐4‐5, specifically Figure 4A without energy minimization and 4B after energy minimization. In Figure 4C the trend of the RMSD values before and after energy minimization of K1‐4‐5 with all the 20 NMR HT2 conformers is reported.

MMF calculations were confirmed by deconvolution of the ATR‐FTIR spectra of K1 and K145 peptides in solid state. In particular, deconvolution of the band related to amide I (1600–1700 cm^–1^) was performed, through which we were able to distinguish overlapping bands due to various protein segments with different secondary structures [27, 28].

**FIGURE 5:**
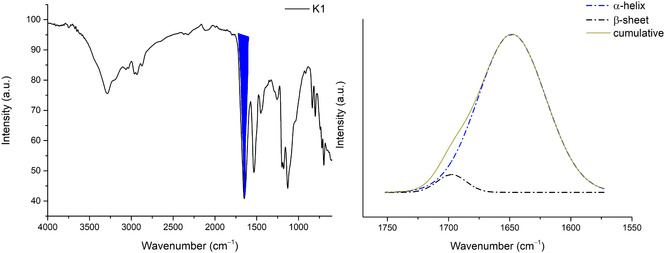
IR spectra of K1 and deconvolution of the band related to amide I (blue region).

Figure 5 shows the spectrum of K1 and the deconvolution of the band related to amide I, highlighted in blue. From the deconvolution, we noted that the α‐helical component is 95% and the remaining part (5%) is attributable to the β‐sheet component [29] in line with the results obtained from MMF studies.

**FIGURE 6:**
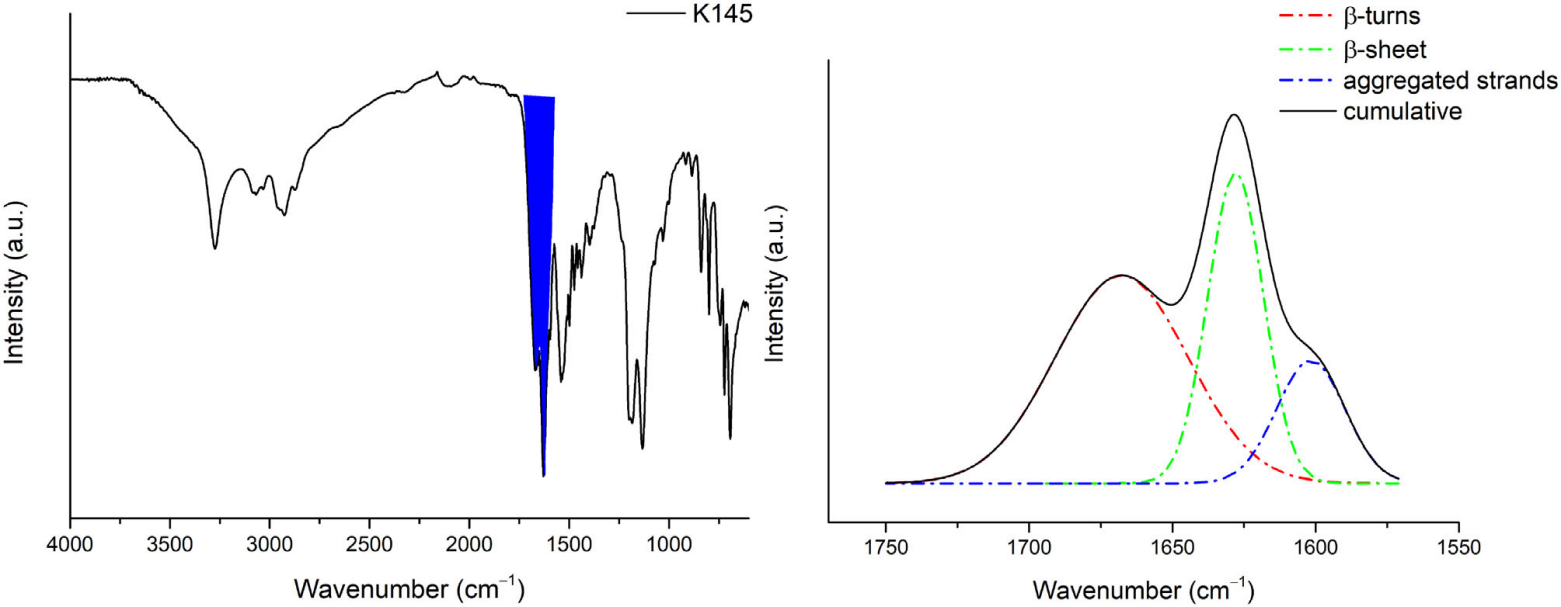
IR spectra of K145 and deconvolution of the band related to amide I (blue region).

The same workflow was performed on the K1‐4‐5 peptide (Figure 6), the deconvolution of which shows the presence of several components, namely: 52% β‐turns, 32% β‐sheet, and 16% aggregated strains [29] as predicted by MMF calculations. It is important to note that the sequence modifications introduced here (Arg→Lys substitutions) were intentionally conservative. This allowed us to isolate and evaluate the predictive power of the MMF approach and the effect of covalent chelation without introducing unreliable sequence‐dependent activity shifts.

To evaluate the antimicrobial potential of our rationally designed α‐helical peptides, we tested HT2 and its two analogs, K1 and K1‐4‐5, both alone and in combination with allomaltol, against three representative bacterial strains: *Staphylococcus aureus* USA300 (methicillin‐resistant), *Klebsiella pneumoniae* ATCC 700603, and *Escherichia coli* ATCC 25922. The results, reported in Table 1, demonstrate that peptide HT2 alone exhibits potent antimicrobial activity against *S. aureus* (MIC = 18.75 μM), moderate activity against *E. coli* (MIC = 75.00 μM), and limited activity against *K. pneumoniae* (MIC = 150.00 μM).

**TABLE 1:** MICs, expressed in μM, of the tested compounds and their combinations against three bacterial strains: *Staphylococcus aureus* USA300, *Klebsiella pneumoniae* ATCC 700603, and *Escherichia coli* ATCC 25922.

COMPOUNDS	*S. aureus* USA300	*K. pneumoniae* 700603	*E. coli* 25922
**HT2**	18.75	150.00	75.00
**Allomaltol**	300.00	300.00	300.00
**K1**	300.00	300.00	300.00
**K1‐allomaltol 1:1**	75.00	300.00	150.00
**K1‐allomaltol 1:2**	75.00	300.00	150.00
**K1‐4‐5**	300.00	150.00	75.00
**K1‐4‐5‐allomaltol 1:1**	37.50	75.00	37.50
**K1‐4‐5‐allomaltol 1:2**	37.50	75.00	37.50
**K 1‐4‐5‐A**	18.75	300.00	75.00

By comparison, peptide K1—differing from HT2 by a single substitution of the *N*‐terminal arginine with lysine—was inactive at all tested concentrations (MIC > 300 μM), suggesting that the guanidinium group of arginine may be essential for interaction with the bacterial membrane or for maintaining a favorable amphipathic *α*‐helical structure. This observation is consistent with previous studies reporting that arginine residues confer enhanced membrane permeabilization through bidentate hydrogen bonding and improved electrostatic interactions with negatively charged bacterial membranes [30, 31, 32].

Interestingly, the analog K1‐4‐5, in which both Arg4 and Arg5 were replaced with lysines, retained partial activity (MIC = 150 μM vs *K. pneumoniae* and 75 μM vs *E. coli*), indicating that the position and number of cationic residues influence not only the overall charge, but also the spatial distribution, which may in turn impact membrane binding and insertion dynamics.

The most impressive and useful result emerges from the coadministration of peptides with allomaltol. Allomaltol alone showed only weak antimicrobial activity (MIC = 300 μM across all strains), but its combination with K1 or K1‐4‐5 significantly enhanced antibacterial efficacy, particularly in the case of K1‐4‐5. The K1‐4‐5–allomaltol combination exhibited MIC values as low as 37.5 μM against both *S. aureus* and *E. coli* and 75 μM against *K. pneumoniae*, suggesting a clear synergistic effect. The synergy may arise from a dual mechanism: while the peptide exerts a membranolytic action, the allomaltol's well‐known metal chelation capabilities [33] contribute to the disruption of the activity of essential bacterial enzymes and metabolic pathways [34]. The enhanced activity observed with the 1:2 ratio (peptide:allomaltol) compared to 1:1 is marginal (Table 1), indicating that the synergy does not scale linearly with allomaltol concentration and that a threshold concentration might suffice to elicit the chelation‐based mechanism.

The markedly enhanced potency of K1‐4‐5A relative to K1‐4‐5 can be rationalized through a cooperative dual‐hit mechanism. MMF predictions and ATR‐FTIR analyses indicate that the covalently attached chelating group introduces only local perturbations, preserving the amphipathic fold required for membrane interaction. Similar behavior has been reported for α‐helical AMPs where structural modifications modulate activity without abolishing membrane affinity [35, 36]. Once the peptide domain anchors the conjugate at the cell surface, the tethered chelator may induce localized Fe(III) withdrawal, a process known to sensitize bacteria to antimicrobial agents [37].

These findings support the hypothesis that combining membrane‐disrupting peptides with small molecules possessing complementary modes of action—such as chelation or oxidative stress induction—can overcome limitations of peptide monotherapy and may reduce the likelihood of resistance development [38, 39]. Furthermore, the observed improvements in activity of analog K1‐4‐5 over K1 highlight the importance of carefully balancing the position, type, and number of charged residues during peptide design to maintain optimal amphiphilicity, conformation, and structural stability.

**FIGURE 7:**
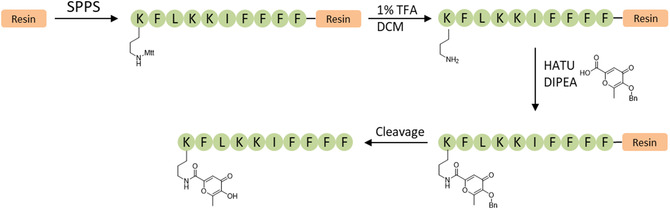
Schematic synthesis of K1‐4‐5‐A.

To further examine the association of amphipathic peptides structures and chelating agents, a third molecule K1‐4‐5 was covalently modified with a chelating agent (K1‐4‐5‐A Figure 2) and was designed and synthesized (Figure 7). The synthesis was performed using Fmoc‐based solid‐phase peptide synthesis (SPPS) on a Rink amide resin, affording a C‐terminally amidated product. Peptide elongation was performed using standard coupling conditions with DIC/Oxyma‐mediated couplings of Fmoc‐protected amino acids bearing appropriate side chain protecting groups. The synthesis was concluded with Fmoc‐Lys(Mtt)‐OH, which contains an orthogonally removable protecting group on the ε‐amino side chain, allowing for selective postsynthetic functionalization. After assembly of the full sequence, the *N*‐terminal Fmoc group was left intact to prevent undesired modifications, and the Mtt (4‐methyltrityl) protecting group was selectively removed using 1% TFA in DCM, thus exposing the ε‐amino group on the terminal lysine side chain. The free amine was then conjugated to 3‐(Benzyloxy)‐6‐methyl‐4‐oxo‐4*H*‐pyran‐2‐carboxylic acid (i.e. an analog of allomaltol, with comparable chelating properties) via amide bond formation using HATU and DIPEA as coupling reagents in DMF under mild conditions. Subsequently, the *N*‐terminal Fmoc‐protecting group on the terminal lysine was removed by treatment with 20% piperidine in DMF, uncaging the free amino terminus. Finally, global cleavage and side chain deprotection were performed using a TFA/phenol/H_2_O/ TIPS (90:5:2.5:2.5, v/v) cocktail, yielding the fully deprotected K1‐4‐5‐A.

Among the tested compounds, K1‐4‐5‐A demonstrates a distinct antimicrobial profile characterized by selective and enhanced activity against *Staphylococcus aureus* USA300, with MIC of 18.75 μM, matching the efficacy of the reference compound HT2. This result is particularly notable when compared to the parent compound K1‐4‐5, which showed no significant activity against *S. aureus* (MIC = 300 μM), suggesting that the structural modification leading to K1‐4‐5‐A confers a substantial improvement in potency against this Gram‐positive strain. Overall, this study highlights the potential of rationally designed *α*‐helical peptides, especially when used in combination with synergistic adjuvants, as promising candidates in the fight against antibiotic‐resistant bacteria. All results are summarized in Table 1. The observed synergistic activity between the target peptides and allomaltol can be rationalized through a dual mechanism. The peptide portion acts as a membrane‐disruptive agent, while the chelating residue removes Fe(III) from the extracellular milieu, impairing essential bacterial metabolic processes. Importantly, both the noncovalent and covalent (K1‐4‐5‐A) strategies preserve the conformation of the peptide in its α‐helical structure, in agreement with previous studies [40]. This dual‐target strategy can be adopted to enhance the overall potency of peptides and provides a promising template for multimodal antimicrobial development. Although the K1‐4‐5A analog incorporates a non‐natural chelating moiety, several lines of evidence suggest that its physicochemical profile is compatible with a favorable therapeutic index. Short α‐helical AMPs with comparable length and net charge typically exhibit limited hemolysis at concentrations well above their MIC values [35, 41]. Moreover, toxicity of synthetic AMPs correlates primarily with excessive hydrophobicity, rather than with local side‐chain modifications [42]. In addition, hydroxypyridinone‐based chelators analogous to the residue used here have undergone substantial preclinical evaluation and demonstrated acceptable biocompatibility [16, 43]. Although these data support the plausibility of a favorable safety window for K1‐4‐5A, dedicated studies will be required and will represent a focus of future work. Also as HT2 was already reported as cytotoxic, the cytotoxicity of K1 and K1‐4‐5 was calculated using ToxinPred. ToxinPred is an in silico method, which is developed to predict and design toxic/nontoxic peptides. The server was able to identify the nontoxicity of HT2 (Figure S5, Supporting Information) as well as calculate noncytotoxicity for the others K1 and K1‐4‐5 (Figures 6 and 7).

## 3 Experimental Section

### 3.1 General Information

All chemicals were purchased from Merck or Fluorochem and used as received. Organic solvents were removed under reduced pressure at 40°C. Synthetic‐purity solvents were used. Silica gel 60 F254, Merck precoated aluminum sheets were employed for thin‐layer chromatography and spots were visualized under UV light. Low‐resolution mass spectra were obtained on a Thermofisher LCQ DECA XP ion trap mass spectrometer or a Waters‐Micromass ZQ‐Single quadrupole mass spectrometer. High‐resolution mass spectra were obtained on a Thermo Exactive Orbitrap MS operating in ESI^+^ mode (Figures S1–S4, Supporting Information). Isotopic distributions were calculated using MolecularWeight Calculator version 6.46. Analytical reverse ‐phase (RP)‐high‐performance liquid chromatography (HPLC) was carried out on a HP1050 HPLC system equipped with an autosampler, a quaternary pump, and a diode‐array detector (DAD). The HPLC column was a Zorbax SB C‐18 2.1 mm _ 10 cm (particle size 5 micrometer). The employed flow rate was 0.3 mL/min and the eluents were monitored at wavelengths between 210 and 280 nm. A linear gradient of mobile phase B (acetonitrile (ACN)) containing 0.1% trifluoroacetic acid (TFA) over mobile phase A (0.1% TFA in water) from 0 to 90% B in 31 min was performed. Data were collected and analyzed using Origin software. FTIR–ATR analyses were conducted using an FTIR Agilent Cary 630 equipped with ATR sampling module. Thin films of all samples were placed on the ATR crystal and pressed. The results were derived from 512 scans acquired in the range of 4000–500 cm^−1^ with a resolution of 2 cm^−1^ at room temperature. The toxicity of peptides was estimated using ToxinPred (https://webs.iiitd.edu.in/raghava/toxinpred/index.html), this server was specifically developed for predicting toxicity of peptides (Figures S5–S7, Supporting Information).

### 3.2 Synthesis

#### 3.2.1 Synthesis of HT2, K1, and K1‐4‐5

The linear sequences [HT2 = ARG‐PHE‐LEU‐ARG–ARG‐ILE‐PHE–PHE‐PHE–PHE; K1 = LYS‐PHE‐LEU‐ARG–ARG‐ILE‐PHE–PHE‐PHE–PHE; K1‐4‐5 = LYS‐PHE‐LEU‐LYS–LYS‐ILE‐PHE–PHE‐PHE–PHE] were synthesized using a standard Fmoc SPPS approach. All the Fmoc (9‐fluorenylmethyloxycarbonyl)‐protected amino acids used were equipped with classical acid‐labile side‐chain protecting groups. Fmoc‐Rink Amide AM resin (Iris Biotech, loading 0.63 mmol/g, 0.1 g) was used as the solid support to obtain C‐terminally amidated peptides. The resin was first swollen in dry DCM (3 mL) for 30 min at room temperature. Following swelling, the Fmoc‐protecting group of the resin was removed by treatment with 20% piperidine in DMF for 20 min. The resin was then thoroughly washed with DMF (3 × 5 mL) and DCM (3 × 5 mL). For the peptides elongation a manual Fmoc SPPE protocol was used: in each step, the resin was reacted with 4 equiv. of Fmoc‐protected amino acid, 4 equiv. of oxyma (Ethyl cyanohydroxyiminoacetate), and 4 equiv. of DIC (*N*,*N*′‐Diisopropylcarbodiimide) in dry DMF (2 mL). The reaction mixture was allowed to mix in the reactor for 4 h at room temperature, followed by Fmoc deprotection with 20% piperidine in DMF for 20 min. Upon completion of the synthesis, the peptides were cleaved from the resin using a cleavage cocktail composed of TFA/phenol/water/TIPS (90:5:2.5:2.5). The mixture was stirred at room temperature for 1 h. The cleavage solution was then concentrated under reduced pressure, and the crude peptides were precipitated in cold diethyl ether. The precipitates were collected by centrifugation and dried under vacuum. Crude products were analyzed by RP‐HPLC and mass spectrometry.

The final compounds were purified via preparative HPLC/UV with a Gemini 5 μ C18 110A/AXIA column (100 × 30 mm × 5 micron), using 0.1% TFA in H_2_O as eluent A and 0.1% TFA in ACN as eluent B. The elution program used a linear gradient of 0%–40% of the eluent B in 60 min. The detection wavelength was 281 nm, and the flow rate was 15 mL min^−1^. The isolated fractions were further analyzed by analytical HPLC‐DAD. The pure fractions were then lyophilized and obtained as white solid (yield 60%–70%, for the last step after preparative purification). **HT2** Calculated ESI^+^ [M + 2H]^2+^: 724.43. Measured ESI^+^ [M + 2H]^2+^: 724.68 (Figure S1, Supporting Information). Calculated Elemental Analysis: C, 62.22; H, 7.38; N, 19.35; O, 11.05. Measured Elemental Analysis: C, 62.13; H, 7.51; N, 19.32; O, 11.04. **K1** Calculated ESI^+^ [M + 2H]^2+^: 710.42. Measured ESI^+^ [M + 2H]^2+^: 710.73 (Figure S2, Supporting Information). Calculated Elemental Analysis: C, 63.45; H, 7.53; N, 17.76; O, 11.27. Measured Elemental Analysis: C, 63.31; H, 7.72; N, 17.72; O, 11.24. **K1‐4‐5** Calculated ESI^+^ [M + 2H]^2+^: 682.42. Measured ESI^+^ [M + 2H]^2+^: 682.64 (Figure S3, Supporting Information). Calculated Elemental Analysis: C, 66.05; H, 7.83; N, 14.38; O, 11.73. Measured Elemental Analysis: C, 65.96; H, 7.97; N, 14.36; O, 11.71.

#### 3.2.2 Synthesis of K1‐4‐5‐A

3‐(Benzyloxy)‐6‐methyl‐4‐oxo‐4H‐pyran‐2‐carboxylic acid was synthesized following a previously reported procedure in the literature [44]. After completion of the SPPS of K1‐4‐5, the *N*‐terminal Fmoc group of Fmoc‐L‐lysine(Mtt)‐OH was retained to prevent undesired modifications. The 4‐methyltrityl (Mtt) protecting group on the *ε*‐amino side chain was selectively removed by treating the resin with 1% TFA in DCM) (2 × 2 mL, 15 min each). The resin was washed with DMF (3 × 5 mL) and DCM (3 × 5 mL) and then dried under vacuum. The resulting resin‐bound peptide (100 mg), bearing a free *ε*‐amino group on the terminal lysine, was suspended in dry DMF (2 mL). Separately, 3‐(benzyloxy)‐6‐methyl‐4‐oxo‐4*H*‐pyran‐2‐carboxylic acid (4 eq., 0.252 mmol, 65.58 mg) was dissolved in DMF (1 mL), followed by the addition of 1‐[bis(dimethylamino)methylene]‐1*H*‐1,2,3‐triazolo[4,5‐b]pyridinium 3‐oxid hexafluorophosphate (HATU, 1.5 eq., 0.378 mmol, 143.73 mg) and *N*,*N*‐diisopropylethylamine (DIPEA, 5 eq., 1.26 mmol, 162.86 mg, 0.214 mL). The solution was stirred at room temperature for 5 min and then added to the resin. The suspension was then stirred overnight at room temperature. After coupling, the mixture was filtered and washed with DMF (3 × 5 mL) and DCM (3 x 5 mL). Fmoc deprotection was performed by treatment with 20% piperidine in DMF (2 mL) for 20 min at room temperature. The resin was subsequently washed with DMF (3 × 5 mL) and DCM (3 × 5 mL), and dried under vacuum. Cleavage of the peptide from the resin and simultaneous removal of acid‐labile protecting groups was carried out using a cleavage cocktail consisting of TFA/phenol/water/TIPS in a 90:5:2.5:2.5 (v/v/v/v) ratio (1 mL). The mixture was stirred at room temperature for 24 h. The cleavage solution was filtered and concentrated under reduced pressure. The crude product was precipitated by addition of cold diethyl ether (10 mL), collected by centrifugation (5300 rpm, 10 min), and dried under vacuum. The product was analyzed by RP‐HPLC and electrospray ionization mass spectrometry. The final compound was purified via preparative HPLC/UV with a Gemini 5μ C18 110A/AXIA column (100 × 30 mm × 5 micron), using 0.1% TFA in H_2_O as eluent A and 0.1% TFA in ACN as eluent B. The elution program used a linear gradient of 0%–40% of the eluent B in 60 min. The detection wavelength was 281 nm, and the flow rate was 15 mL min^−1^. The isolated fractions were further analyzed by analytical HPLC‐DAD. The pure fractions were then lyophilized and obtained as white solid (yield 62%, for the last step after preparative purification). Calculated ESI^+^ [M + 2H]^2+^: 758.42. Measured ESI^+^ [M + 2H]^2+^: 758.85 (Figure S4, Supporting Information). Calculated Elemental Analysis: C, 64.97; H, 7.31; N, 12.94; O, 14.78. Measured Elemental Analysis: C, 64.80; H, 7.56; N, 12.90; O, 14.74.

### 3.3 Antibacterial Activity

The antibacterial activity was evaluated by determining the minimum inhibitory concentration (MIC) of the compounds. MIC values were obtained against methicillin‐resistant *Staphylococcus aureus* USA300, *Klebsiella pneumoniae* ATCC 700603, and *Escherichia coli* ATCC 25922 using the broth microdilution method. Bacterial strains were cultured overnight in Luria Bertani broth (LB Broth Base Lennox formulation Sigma–Aldrich L‐3022) agar at 37°C overnight. From the overnight cultures a standard 0.5 McF solution was prepared and further diluted to the final inoculum with approximately 1.0 × 10^6^ CFU/mL. For each compound, peptides were initially dissolved in DMSO and subsequently diluted in LB broth to obtain the starting stock solution, ensuring a final DMSO concentration not exceeding 10%. Moreover, serial dilutions were performed, yielding final concentrations ranging from 600 to 9.38 μM. An aliquot of bacterial suspension was subsequently added to each well to reach a final concentration of 1.0 × 10^5^ CFU/mL. Wells containing only the bacterial inoculum served as positive controls, while wells with only LB broth served as negative controls. The microplates were incubated overnight at 37 °C, and the MIC was determined as the lowest concentration at which no visible bacterial growth was observed.

## 4 Conclusion

This study provides the first demonstration that the outcomes of MMFs‐based modeling can prospectively align well with experimentally confirmed secondary structures of bioactive peptides, offering a new predictive framework for reliable peptide design. By employing HT2 as a structural and functional reference, we successfully demonstrated the predictive power and accuracy of MMFs calculations, particularly when validated through comparison with NMR‐derived conformers. The RMSD analysis confirmed the ability of MMFs to reproduce HT2‐like structures with good fidelity, especially following energy minimization. K1 retained the essential cationic nature of the *N*‐terminus while improving structural congruence. K1‐4‐5 further extended this concept by increasing lysine content, preserving helical features. Overall, our findings reinforce the utility of MMFs‐based computational design for guiding the development of bioactive *α*‐helical peptides with tailored properties. The analogs presented here maintain key structural features associated with the antimicrobial function of HT2. Biological evaluation of these peptides revealed potent antimicrobial activity against a panel of clinically relevant bacterial strains, including multidrug‐resistant Gram‐positive and Gram‐negative species. Notably, when these peptides were coadministered with an allomaltol, a metal‐chelating agent, a synergistic enhancement in antimicrobial efficacy was observed. This combinatorial approach exploits a dual mechanism of action: peptide‐mediated membrane disruption and allomaltol‐induced sequestration of essential metal ions, collectively impairing bacterial viability through complementary pathways. The enhanced performance of peptide–allomaltol combinations highlights the therapeutic potential of these hybrid strategies in overcoming resistance mechanisms, particularly in drug‐refractory infections. Another derivative resulting from covalently bonding a chelating agent with the sequence of K1‐4‐5, exploiting a terminal lysine, demonstrates a distinct antimicrobial profile characterized by selective and enhanced activity against *Staphylococcus aureus*, with a MIC of 18.75 μM. Overall, our findings indicate that the integration of MMFs‐based rational design of peptides with synergistic chelating agents adjuvants (like allomaltol) constitutes a promising platform for the development of next‐generation antimicrobial therapeutics, both in coadministration and being covalently bonded. The pronounced selectivity of K1‐4‐5A toward *S. aureus* can be explained by the convergence of two vulnerabilities characteristic of Gram‐positive pathogens. First, the abundance of wall teichoic acids provides a dense anionic matrix that concentrates cationic AMPs at the bacterial surface [45, 46]. Second, *S. aureus* exhibits a stringent dependence on iron and manganese homeostasis for respiration, virulence regulation, and oxidative‐stress defense [47]. Hydroxypyridinone‐based chelators structurally related to the one employed here also display direct antistaphylococcal activity [18]. Together, these elements support a dual‐mechanism model in which electrostatic targeting enhances the impact of localized metal deprivation in *S. aureus.* Future work will focus on in vivo validation, toxicity profiling, and formulation strategies to advance the overall approach, as well as these candidates toward clinical translation. In summary, this study validates the MMF method as a predictive and structurally faithful modeling framework for α‐helical peptides, while demonstrating that the covalent integration of chelating moieties into peptide structures substantially enhances antimicrobial potency. Although absolute MIC values resulted moderate within the molecular series reported herein, the combined computational–chemical approach establishes a promising blueprint for rational, mechanism‐driven AMP development. Considering the results obtained, we suppose that the most critical aspect may be the destructive interaction, in terms of antimicrobial activity, between the peptide and the chelating agent, which prevents the two components from performing their antimicrobial action to the fullest. In order to improve this approach, other synthetic strategies and/or other chelating agents will be considered. For example, chains that act as spacers between the chelating agent and the peptide will be evaluated to limit destructive interactions between the peptide and the chelating agent and promote synergy between the two systems.

## Supporting Information

Additional supporting information can be found online in the Supporting Information section. **Supporting** **Fig.** **S1:** ESI MS of HT2 [M + 2H]^2+^. **Supporting** **Fig.** **S2:** ESI MS of K1 [M + 2H]^2+^. **Supporting** **Fig.** **S3:** ESI MS of K1‐4‐5 [M + 2H]^2+^. **Supporting** **Fig.** **S4:** ESI MS of K1‐4‐5‐A [M + 2H]^2+^. **Supporting** **Fig.** **S5:** HT2 data from ToxinPred. **Supporting** **Fig.** **S6:** K1 data from ToxinPred. **Supporting** **Fig.** **S7:** K1‐4‐5 data from ToxinPred.

## Author Contributions

**Vincenzo Patamia**: writing – review & editing, writing – original draft, methodology, formal analysis, data curation. **Erika Saccullo**: methodology, investigation, data curation. **Michele Larocca**: writing – review & editing, writing – original draft, formal analysis, data curation, methodology, software. **Virginia Fuochi**: writing – review & editing, methodology, investigation. **Salvatore Furnari**: methodology, investigation, data curation. **Pio Maria Furneri**: writing – review & editing, validation, formal analysis. **Agostino Cilibrizzi**: writing – review & editing, writing – original draft, formal analysis. **Giuseppe Floresta**: writing – review & editing, writing – original draft, supervision, conceptualization, resources, project administration.

## Conflicts of Interest

The authors declare no conflicts of interest.

## Supporting information

Supplementary Material

## Data Availability

The data that support the findings of this study are available from the corresponding author upon reasonable request.

## References

[1] P. M. Furneri , G. P. Petronio , V. Fuochi , S. Cupri , R. Pignatello , Nanostructures for Drug Delivery, ed. E. Andronescu and A. M. Grumezescu (Elsevier, 2017), 697–748.

[2] R. Hadianamrei , M. A. Tomeh , S. Brown , J. Wang , X. Zhao , “Rationally Designed Short Cationic α‐Helical Peptides with Selective Anticancer Activity,” Journal of Colloid and Interface Science 607 (2022): 488–501. 34509120 10.1016/j.jcis.2021.08.200

[3] C. Zagni , V. Patamia , S. Dattilo , et al., “Supramolecular Biomaterials as Drug Nanocontainers with Iron Depletion Properties for Antimicrobial Applications,” Materials Advances 5 (2024): 3675–3682.

[4] Z. Tang , W. Jiang , S. Li , et al., “Design and Evaluation of Tadpole‐Like Conformational Antimicrobial Peptides,” Communications Biology 6 (2023): 1177. 37980400 10.1038/s42003-023-05560-0 PMC10657444

[5] V. Patamia , C. Zagni , R. Fiorenza , et al., “Total Bio‐Based Material for Drug Delivery and Iron Chelation to Fight Cancer through Antimicrobial Activity,” Nanomaterials 13 (2023): 2036. 37513047 10.3390/nano13142036 PMC10384306

[6] V. Patamia , E. Saccullo , F. Magaletti , et al., “Nature‐Inspired Innovation: Alginic–Kojic Acid Material for Sustainable Antibacterial and Carbon Dioxide Fixation,” International Journal of Biological Macromolecules 277 (2024): 134514. 39111504 10.1016/j.ijbiomac.2024.134514

[7] V. Patamia , E. Saccullo , V. Fuochi , et al., “Developing Advanced Antibacterial Alginic Acid Biomaterials through Dual Functionalization,” ACS Applied Bio Materials 7 (2024): 6932–6940. 10.1021/acsabm.4c01034 39253768

[8] V. Patamia , D. Gentile , R. Fiorenza , et al., “Nanosponges Based on Self‐Assembled Starfish‐Shaped Cucurbit[6]urils Functionalized with Imidazolium Arms,” Chemical Communications 57 (2021): 3664–3667. 33725066 10.1039/d1cc00990g

[9] X. Ma , Q. Wang , K. Ren , et al., “A Review of Antimicrobial Peptides: Structure, Mechanism of Action, and Molecular Optimization Strategies,” Fermentation 10 (2024): 540.

[10] Y. Huan , Q. Kong , H. Mou , H. Yi , “Antimicrobial Peptides: Classification, Design, Application, and Research Progress in Multiple Fields,” Frontiers in Microbiology 11 (2020): 2020. 33178164 10.3389/fmicb.2020.582779 PMC7596191

[11] S. Lohan , A. G. Konshina , R. G. Efremov , I. Maslennikov , and K. Parang , “Structure-Based Rational Design of Small α‐Helical Peptides with Broad–Spectrum Activity against Multidrug–Resistant Pathogens,” Journal of Medicinal Chemistry 66 (2023): 855–874. 36574364 10.1021/acs.jmedchem.2c01708 PMC9841524

[12] A. P. Yakimov , A. S. Afanaseva , M. A. Khodorkovskiy , M. G. Petukhov , “Design of Stable α‐Helical Peptides and Thermostable Proteins in Biotechnology and Biomedicine,” Acta Naturae 8 (2016): 70–81. 28050268 PMC5199208

[13] M. Failla , G. Floresta , V. Abbate , “Peptide‐Based Positron Emission Tomography Probes: Current Strategies for Synthesis and Radiolabelling,” RSC Medicinal Chemistry 14 (2023): 592–623. 37122545 10.1039/d2md00397j PMC10131587

[14] M. Larocca , G. Floresta , D. Verderese , A. Cilibrizzi , “Main Mechanical Forces‐Chemical Interactions’ Interplay as a Tool to Elucidate Folding Mechanisms,” Peptide Science 116 (2024): e24365.

[15] M. Larocca , G. Floresta , D. Verderese , A. Cilibrizzi , “Dominant Chemical Interactions Governing the Folding Mechanism of Oligopeptides,” International Journal of Molecular Sciences 25 (2024): 9586. 39273531 10.3390/ijms25179586 PMC11395422

[16] M. E. Faure , A. Cilibrizzi , V. Abbate , K. D. Bruce , and R. C. Hider , “Effect of Iron Chelation on Anti‐Pseudomonal Activity of Doxycycline,” International Journal of Antimicrobial Agents 58 (2021): 106438. 34547423 10.1016/j.ijantimicag.2021.106438 PMC8617590

[17] K. Itoh , H. Tsutani , Y. Mitsuke , H. Iwasaki , “Potential Additional Effects of Iron Chelators on Antimicrobial‐Impregnated Central Venous Catheters,” Frontiers in Microbiology 14 (2023): 1210747. 37608951 10.3389/fmicb.2023.1210747 PMC10442153

[18] F. P. Nocera , G. Iovane , L. De Martino , B. E. Holbein , “Antimicrobial Activity of the Iron‐Chelator, DIBI, against Multidrug-Resistant Canine Methicillin–Susceptible Staphylococcus pseudintermedius: A Preliminary Study of Four Clinical Strains,” Pathogens 11 (2022): 656. 35745511 10.3390/pathogens11060656 PMC9227175

[19] M. I. Hood and E. P. Skaar , “Nutritional Immunity: Transition Metals at the Pathogen-Host Interface,” Nature Reviews Microbiology 10 (2012): 525–537. 22796883 10.1038/nrmicro2836 PMC3875331

[20] V. Fuochi , F. Salvatore , T. Laura , C. Maddalena , and P. M. Furneri , “Therapies in Preclinical and in Early Clinical Development for the Treatment of Urinary Tract Infections: From Pathogens to Therapies,” Expert Opinion on Investigational Drugs 33 (2024): 677–698. 38700945 10.1080/13543784.2024.2351509

[21] M. Larocca , F. Foglia , A. Cilibrizzi , “Principles that Rule the Calculation of Dihedral Angles in Secondary Structures: the cases of an α‐helix and a β‐sheet,” Biochemistry 58 (2019): 1032–1037. 30719916 10.1021/acs.biochem.8b01101

[22] M. Larocca , G. Floresta , A. Cilibrizzi , “Dihedral Angle Calculations to Elucidate the Folding of Peptides through its Main Mechanical Forces,” Journal of Molecular Structure 1229 (2021): 129802. 10.1021/acs.biochem.8b01101 30719916

[23] M. Larocca , “Polypeptides Folding: Rules for the Calculation of the Backbone Dihedral Angles ϕ Starting from the Amino Acid Sequence,” preprint, ChemRxiv 2020. 10.26434/chemrxiv.12000132.v2.

[24] S. Ramachandran , P. Kota , F. Ding , and N. V. Dokholyan , “Automated Minimization of Steric Clashes in Protein Structures,” Proteins 79 (2011): 261–270. 21058396 10.1002/prot.22879 PMC3058769

[25] W. Humphrey , A. Dalke , and K. Schulten , “VMD: Visual Molecular Dynamics,” Journal of Molecular Graphics 14 (1996): 33–38. 8744570 10.1016/0263-7855(96)00018-5

[26] L. Zhan , J. Z. Y. Chen , and W.‐K. Liu , “Conformational Study of Met‐Enkephalin Based on the ECEPP Force Fields,” Biophysical Journal 91 (2006): 2399–2404. 16829555 10.1529/biophysj.106.083899 PMC1562380

[27] W. K. Surewicz , H. H. Mantsch , D. Chapman , “Determination of Protein Secondary Structure by Fourier Transform Infrared Spectroscopy: A Critical Assessment,” Biochemistry 32 (1993): 389–394. 8422346 10.1021/bi00053a001

[28] H. Fabian , C. Schultz , D. Naumann , O. Landt , U. Hahn , W. Saenger , “Secondary Structure and Temperature‐Induced Unfolding and Refolding of Ribonuclease T1 in Aqueous Solution: A Fourier Transform Infrared Spectroscopic Study,” Journal of Molecular Biology 232 (1993): 967–981. 8355280 10.1006/jmbi.1993.1442

[29] K. Bagińska , J. Makowska , W. Wiczk , F. Kasprzykowski , and L. Chmurzyński , “Conformational Studies of Alanine‐Rich Peptide Using CD and FTIR Spectroscopy,” Journal of Peptide Science 14 (2008): 283–289. 17918765 10.1002/psc.923

[30] P. F. Almeida , A. Pokorny , “Mechanisms of Antimicrobial, Cytolytic, and Cell‐Penetrating Peptides: From Kinetics to Thermodynamics,” Biochemistry 48 (2009): 8083–8093. 19655791 10.1021/bi900914g PMC2774275

[31] W. Li , F. Separovic , N. M. O’Brien‐Simpson , J. D. Wade , “Chemically Modified and Conjugated Antimicrobial Peptides against Superbugs,” Chemical Society Reviews 50 (2021): 4932–4973. 33710195 10.1039/d0cs01026j

[32] V. Fuochi , A. Rosato , R. Emma , P. M. Furneri , “Colistin and Kanamycin Together in Association with Coridothymus Capitatus to Enhance Their Antimicrobial Activity and Fight Multidrug‐Resistance Pathogens,” Biointerface Research in Applied Chemistry 11 (2021): 8608–8625.

[33] A. Cilibrizzi , V. Abbate , Y.‐L. Chen , Y. Ma , T. Zhou , and R. C. Hider , “Hydroxypyridinone Journey into Metal Chelation,” Chemical Reviews 118 (2018): 7657–7701. 30033720 10.1021/acs.chemrev.8b00254

[34] L. Grassi , G. Maisetta , S. Esin , and G. Batoni , “Combination Strategies to Enhance the Efficacy of Antimicrobial Peptides against Bacterial Biofilms,” Frontiers in Microbiology 8 (2017): 2409. 29375486 10.3389/fmicb.2017.02409 PMC5770624

[35] Y. Chen , C. T. Mant , S. W. Farmer , R. E. Hancock , M. L. Vasil , R. S. Hodges , “Rational Design of Alpha‐Helical Antimicrobial Peptides with Enhanced Activities and Specificity/Therapeutic Index,” Journal of Biological Chemistry 280 (2005): 12316–12329. 15677462 10.1074/jbc.M413406200 PMC1393284

[36] Z. Jiang , A. I. Vasil , J. D. Hale , R. E. Hancock , M. L. Vasil , R. S. Hodges , “Effects of Net Charge and the Number of Positively Charged Residues on the Biological Activity of Amphipathic Alpha-Helical Cationic Antimicrobial Peptides,” Biopolymers 90 (2008): 369–383. 18098173 10.1002/bip.20911 PMC2761230

[37] E. P. Skaar , “The Battle for Iron between Bacterial Pathogens and Their Vertebrate Hosts,” PLoS Pathogens 6 (2010): e1000949. 20711357 10.1371/journal.ppat.1000949 PMC2920840

[38] N. Mookherjee , M. A. Anderson , H. P. Haagsman , D. J. Davidson , “Antimicrobial Host Defence Peptides: Functions and Clinical Potential,” Nature Reviews Drug Discovery 19 (2020): 311–332. 32107480 10.1038/s41573-019-0058-8

[39] M. Mahlapuu , J. Håkansson , L. Ringstad , C. Björn , “Antimicrobial Peptides: An Emerging Category of Therapeutic Agents,” Frontiers in Cellular and Infection Microbiology 6 (2016): 194. 28083516 10.3389/fcimb.2016.00194 PMC5186781

[40] R. Bellavita , S. Braccia , L. E. Imbò , et al., “Exploring Fe(III) Coordination and Membrane Interaction of a Siderophore‐Peptide Conjugate: Enhancing Synergistically the Antimicrobial Activity,” Journal of Inorganic Biochemistry 259 (2024): 112658. 38964199 10.1016/j.jinorgbio.2024.112658

[41] Y. Huang , J. Huang , and Y. Chen , “Alpha-Helical Cationic Antimicrobial Peptides: Relationships of Structure and Function,” Protein & Cell 1 (2010): 143–152. 21203984 10.1007/s13238-010-0004-3 PMC4875170

[42] L. E. Uggerhoj , T. J. Poulsen , J. K. Munk , et al., “Rational Design of Alpha‐Helical Antimicrobial Peptides: Do's and Don'ts,” ChemBioChem 16 (2015): 242–253. 25530580 10.1002/cbic.201402581

[43] A. Cilibrizzi , V. Abbate , Y. L. Chen , Y. Ma , T. Zhou , and R. C. Hider , “Hydroxypyridinone Journey into Metal Chelation,” Chemical Reviews 118 (2018): 7657–7701. 30033720 10.1021/acs.chemrev.8b00254

[44] C. V. Credille , B. L. Dick , C. N. Morrison , et al., “Structure-Activity Relationships in Metal‐Binding Pharmacophores for Influenza Endonuclease,” Journal of Medicinal Chemistry 61 (2018): 10206–10217. 30351002 10.1021/acs.jmedchem.8b01363 PMC6249039

[45] T. Koprivnjak , C. Weidenmaier , A. Peschel , J. P. Weiss , “Wall Teichoic Acid Deficiency in Staphylococcus aureus Confers Selective Resistance to Mammalian Group IIA Phospholipase A(2) and Human Beta‐Defensin 3,” Infection and Immunity 76 (2008): 2169–2176. 18347049 10.1128/IAI.01705-07 PMC2346714

[46] C. Weidenmaier , A. Peschel , “Teichoic Acids and Related Cell‐Wall Glycopolymers in Gram-Positive Physiology and Host Interactions,” Nature Reviews Microbiology 6 (2008): 276–287. 18327271 10.1038/nrmicro1861

[47] M. C. van Dijk , R. M. de Kruijff , and P. L. Hagedoorn , “The Role of Iron in Staphylococcus aureus Infection and Human Disease: A Metal Tug of War at the Host‐Microbe Interface,” Frontiers in Cell and Developmental Biology 10 (2022): 857237. 35399529 10.3389/fcell.2022.857237 PMC8986978

