# Supplementary material for: Engineered α‐Helical Peptides with Chelating Agents as Approach to Antibacterial Therapeutics
Source: ChemistryOpen. 2026 Jan 8;15(1):e202500588. doi: 10.1002/open.202500588 (PMC12782638; doi:10.1002/open.202500588)
Supplement: Supplementary file 1 — Supplementary Material [file OPEN-15-e202500588-s001.pdf]

Supporting Information

Engineered α-Helical Peptides with Chelating Agents as Approach to Antibacterial Therapeutics

Vincenzo Patamia [1], Erika Saccullo [1,2], Michele Larocca [3], Virginia Fuochi [2], Salvatore Furnari [2],Pio Maria Furneri [2], Agostino Cilibrizzi [4,5], Giuseppe Floresta [*1]

[1] Department of Drug and Health Sciences, University of Catania, Viale Andrea Doria 6, 95125 Catania, Italy

[2] Department of Biomedical and Biotechnological Sciences (Biometec), University of Catania, Via Santa Sofia 97, 95123 Catania, Italy

[3] Department of Chemistry and Biology "Adolfo Zambelli", University of Salerno, Via Giovanni Paolo II 132, 84084, Fisciano (Salerno), Italy

[4] Institute of Pharmaceutical Science, King's College London, Stamford Street, London SE1 9NH, UK

[5] Centre for Therapeutic Innovation, University of Bath, Bath BA2 7AY, UK

Correspondence: giuseppe.floresta@unict.it

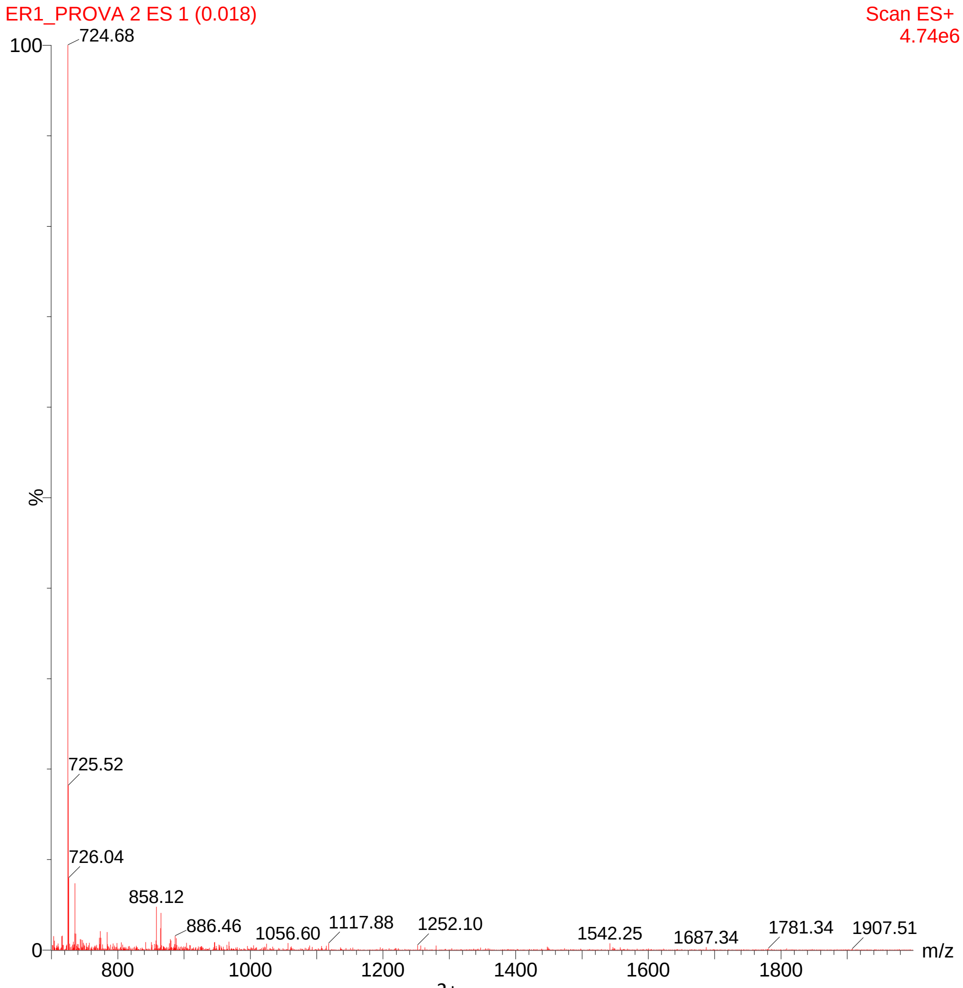

Figure S1. ESI MS of HT2 $[M + 2H]^{2+}$.

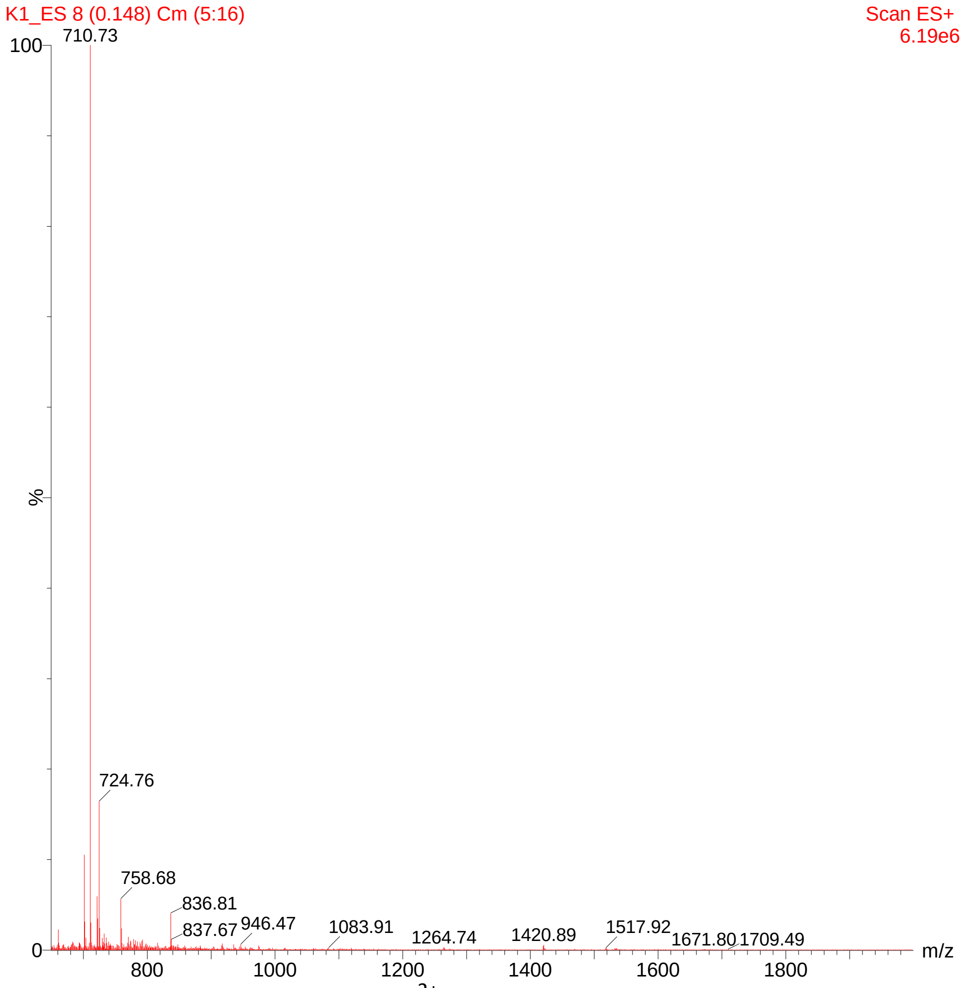

Figure S2. ESI MS of K1 $[M + 2H]^{2+}$.

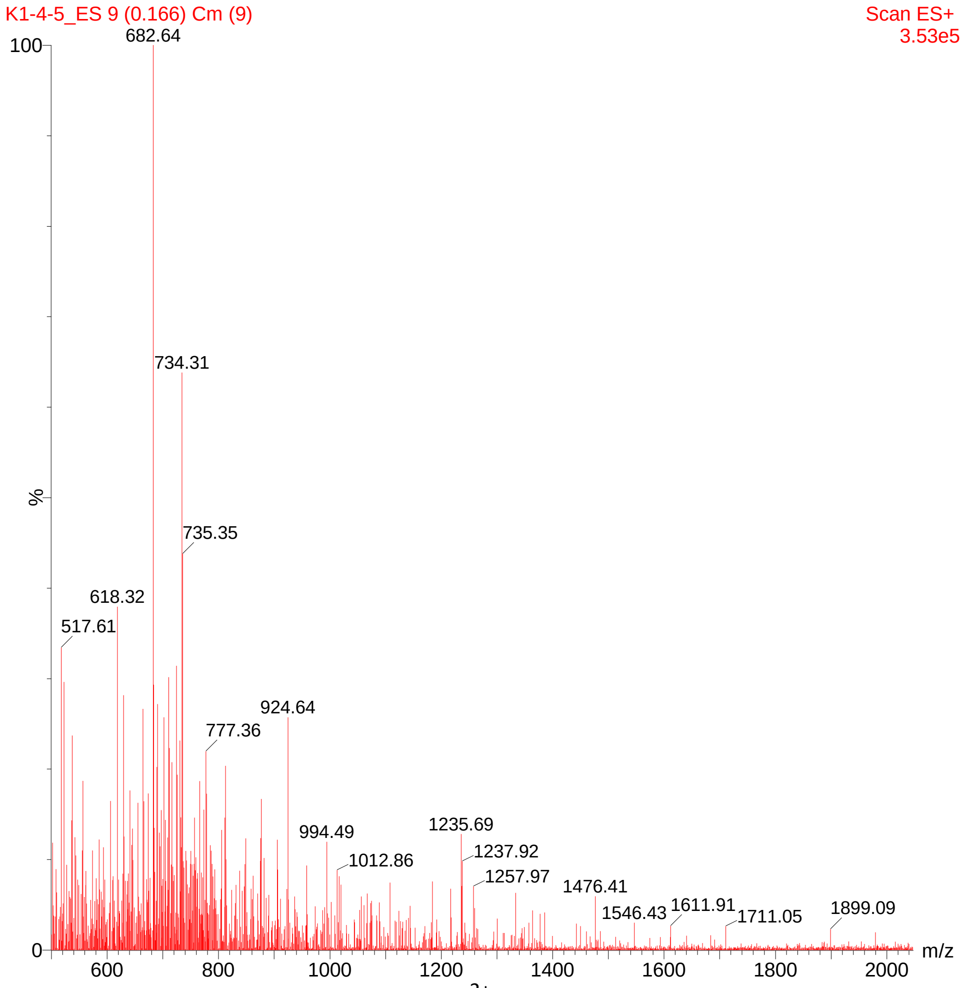

Figure S3. ESI MS of K1-4-5 $[M + 2H]^{2+}$.

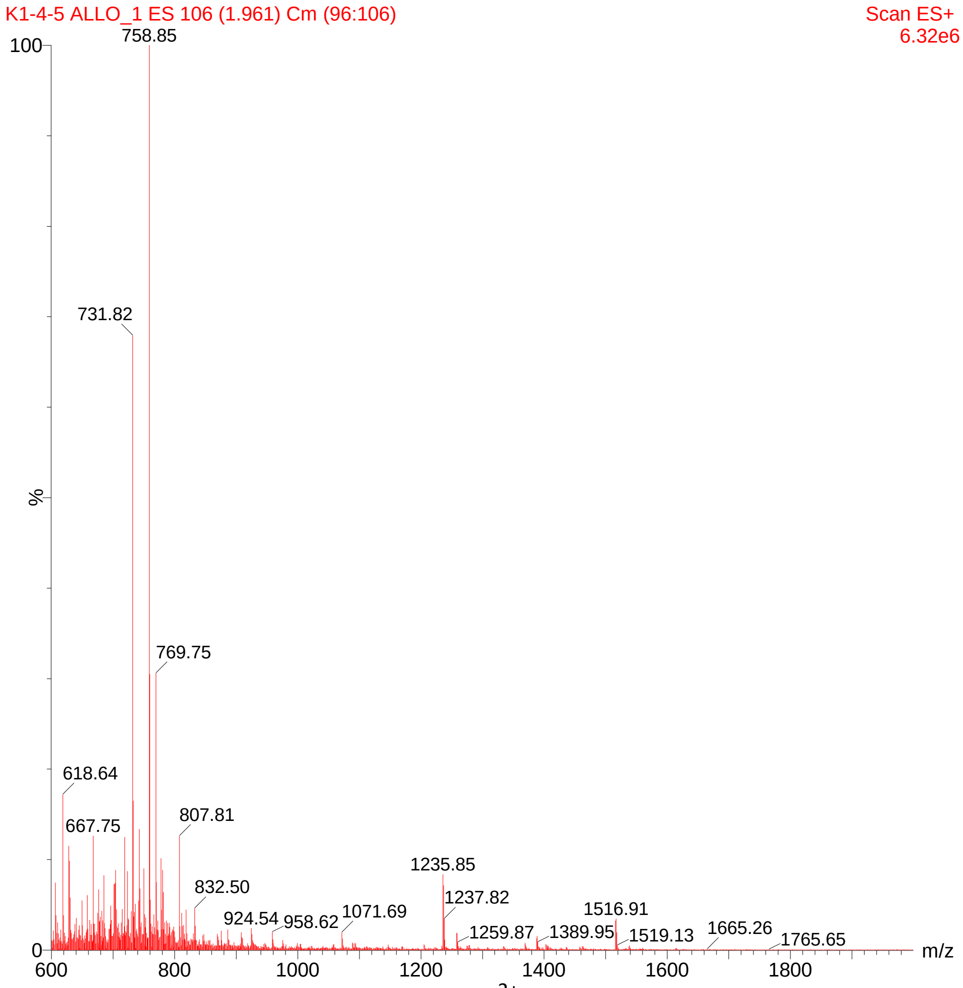

Figure S4. ESI MS of K1-4-5-A $[M + 2H]^{2+}$.

Original Peptide							
Peptide Sequence	Mutation Position	SVM score	Prediction	Hydrophobicity	Hydropathicity	Hydrophilicity	Charge
RFLRRIFFFF	No Mutation	-0.87	Non-Toxin	-0.10	0.88	-0.71	3.00

Figure S5. HT2 data from ToxinPred

Original Peptide							
Peptide Sequence	Mutation Position	SVM score	Prediction	Hydrophobicity	Hydropathicity	Hydrophilicity	Charge
KFLRRIFFFF	No Mutation	-0.82	Non-Toxin	-0.03	0.94	-0.71	3.00

Figure S6. K1 data from ToxinPred

Original Peptide							
Peptide Sequence	Mutation Position	SVM score	Prediction	Hydrophobicity	Hydropathicity	Hydrophilicity	Charge
KFLKKIFFFF	No Mutation	-0.80	Non-Toxin	0.10	1.06	-0.71	3.00

Figure S7. K1-4-5 data from ToxinPred
